# Efficient and Specific Internal Cleavage of a Retroviral Palindromic DNA Sequence by Tetrameric HIV-1 Integrase

**DOI:** 10.1371/journal.pone.0000608

**Published:** 2007-07-11

**Authors:** Olivier Delelis, Vincent Parissi, Hervé Leh, Gladys Mbemba, Caroline Petit, Pierre Sonigo, Eric Deprez, Jean-François Mouscadet

**Affiliations:** 1 LBPA, CNRS UMR8113, Ecole Normale Supérieure de Cachan, Cachan, France; 2 Laboratoire REGER, IFR 66 “Pathologies infectieuses et cancer”, Bordeaux, France; 3 Génétique des Virus, Département des Maladies Infectieuses, Institut Cochin, INSERM U567, CNRS UMR8104, Université René Descartes, Paris, France; Institut Pasteur, France

## Abstract

**Background:**

HIV-1 integrase (IN) catalyses the retroviral integration process, removing two nucleotides from each long terminal repeat and inserting the processed viral DNA into the target DNA. It is widely assumed that the strand transfer step has no sequence specificity. However, recently, it has been reported by several groups that integration sites display a preference for palindromic sequences, suggesting that a symmetry in the target DNA may stabilise the tetrameric organisation of IN in the synaptic complex.

**Methodology/Principal Findings:**

We assessed the ability of several palindrome-containing sequences to organise tetrameric IN and investigated the ability of IN to catalyse DNA cleavage at internal positions. Only one palindromic sequence was successfully cleaved by IN. Interestingly, this symmetrical sequence corresponded to the 2-LTR junction of retroviral DNA circles—a palindrome similar but not identical to the consensus sequence found at integration sites. This reaction depended strictly on the cognate retroviral sequence of IN and required a full-length wild-type IN. Furthermore, the oligomeric state of IN responsible for this cleavage differed from that involved in the 3′-processing reaction. Palindromic cleavage strictly required the tetrameric form, whereas 3′-processing was efficiently catalysed by a dimer.

**Conclusions/Significance:**

Our findings suggest that the restriction-like cleavage of palindromic sequences may be a general physiological activity of retroviral INs and that IN tetramerisation is strongly favoured by DNA symmetry, either at the target site for the concerted integration or when the DNA contains the 2-LTR junction in the case of the palindromic internal cleavage.

## Introduction

Once human immunodeficiency type 1 virus (HIV-1) enters the host cell, its genomic RNA is reverse transcribed to generate a double-stranded linear DNA that is subsequently covalently inserted into host cell chromosomes by integrase (IN). Complete integration involves two spatially and temporally distinct reactions. The first reaction, 3′-processing, occurs in the cytoplasm. The viral DNA is trimmed by IN, releasing a 3′ terminal dinucleotide from each viral DNA extremity, downstream from the canonical subterminal 5′-CA sequence. The second reaction, strand transfer, which takes place after the matured viral DNA has been translocated to the nucleus, results in covalent insertion of the viral DNA into chromosomal DNA. Both reactions require the physiological cofactor, Mg^2+^ [1], [2].

Both 3′-processing and strand transfer reactions can be modelled *in vitro* with the recombinant IN and short oligodeoxynucleotides (ODN) mimicking the extremities of the viral genome. The simultaneous presence in the same complex of the newly exposed 3′-OH extremities of the 3′ processed viral DNA, target DNA and IN allows complete integration to take place *in vitro* [3]–[5]. A specific viral sequence (*att*) is required for 3′-processing, as shown by the inefficiency of this process if the CA sequence is mutated or located more than two nucleotides away from the 3′ end of the DNA [6]–[8]. However, internal cleavage has been reported, but this endonucleolytic activity is not sequence-specific as it occurs independently of the 5′-CA dinucleotide and only in the presence of Mn^2+^.

*In vivo*, 3′-processing and strand transfer occur within the pre-integration complex, a large nucleoprotein complex comprising the viral IN bound to the short cognate sequences located at each end of the long terminal repeat (LTR). The duplicated sequence present at the integration site—the hallmark of retroviral integration—demonstrates the intrinsically symmetric nature of the concerted integration process [9]. The 3′-processing and strand transfer reactions require the formation of an IN oligomer. Both *in vitro* and cellular complementation studies have shown that single inactive IN mutants may combine to form a catalytically competent nucleoprotein, confirming the oligomeric nature of functional IN [1]. Although IN dimers are competent for the 3′-processing reaction, tetramers (dimers of dimers) are required for coupled insertion of the two viral DNA ends [3], [10], [11].

**Figure 1:**
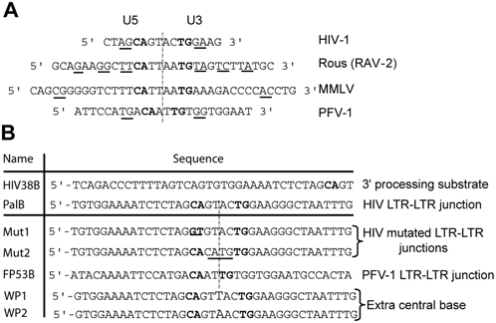
Sequences of retroviral LTR-LTR junctions and oligonucleotide substrates. A) The retroviral sequences found at the LTR-LTR junctions of 2-LTR circles are almost perfect palindromes [15]–[18]. The bases underlined correspond to imperfect palindromic sequences. The 5′-CA dinucleotide (U5-LTR) or its complementary sequence GT (U3-LTR), essential for the 3′-processing reaction, is shown in bold. The vertical dashed line indicates the axis of symmetry. B) Summary of the various DNA substrates used for palindrome cleavage and 3′-processing reactions (only the top strands are shown). The mutations in the Mut1 and Mut2 sequences are underlined.

The 3′-processing reaction is strictly dependent on the presence of the *att* sequence. This is a highly specific reaction as it requires the presence of the CA dinucleotide, at position 3/4 from the DNA 3′-extremity (see sequence HIV38B in Fig. 1B), regardless the cationic cofactor, Mn^2+^ or Mg^2+^. In the presence of Mg^2+^, more positions are involved in the reaction specificity (about the 10 terminal positions) [6], [7]. No such reaction specificity in term of target sequence has been found *in vitro* for the strand transfer reaction. However, large-scale sequencing of *ex vivo* integration sites have revealed a bias towards palindromic sequences, thus providing evidence that target are organised symmetrically rather than randomly [12]–[14]. Thus, tetrameric IN may display significant specificity for symmetric sequences, and the recognition of such sequences in the target may favour the stabilisation of tetramers.

We investigated several palindromic sequences, focusing on possible determinants promoting IN tetramerisation and on the ability of IN to cleave symmetric DNA sequences at internal positions. It is important to note that a symmetric sequence is also present at the LTR-LTR junction of covalently linked viral extremities. Interestingly, this sequence is similar, although not identical, to the palindromic consensus found at the integration sites [12]–[18]. The palindromic sequence in the 2-LTR circles generated in retroviral replication junctions is a general feature of retroviruses (Figure 1A) [15]–[18]. For HIV-1, all strains in infected cells yield in majority the LTR-LTR junction sequence shown in Fig. 1A. The percentage of these junctions in infected cells which matches perfectly with the expected junction sequence (deduced from the sequences of viral DNA extremities) is about 52–58%, the variability in the sequence mostly originates in deletion/insertion at the junction [15], [16]. Two-LTR circles accumulate in cells when IN mutations or the use of strand transfer inhibitors impair integration. They are considered to be dead-end products originating from the cell-mediated ligation of linear viral DNA via the NHEJ pathway [19], [20].

We then tested either palindromic sequences derived from the preferential integration sites or the retroviral sequence present at the LTR-LTR junction. We investigated *in vitro* cleavage conditions and demonstrated, for the first time, that HIV-1 IN cleaves DNA at internal positions, in a sequence-specific manner with the same efficiency as compared to the 3′-processing reaction. This cleavage is symmetric and occurs only in the LTR-LTR palindromic sequence—it mainly occurs at the canonical CA positions on opposite strands and is restricted to the cognate substrate of HIV-1 IN. This activity differs from other IN activities, such as disintegration or 3′-processing, as it is restricted to the full-length tetrameric form of the protein.

## Methods

### Oligonucleotides and plasmids

The sequences of the different ODN substrates are shown in Figure 1B. ODNs were purchased from Eurogentec (Liege, Belgium) and further purified by electrophoresis in a denaturing 18% acrylamide/urea gel. For activity assays, ODNs were radiolabelled with T4 polynucleotide kinase (Biolabs) and γ[^32−^P]ATP (3000 Ci/mmol) (Amersham), and purified on a Sephadex G-10 column (GE Healthcare). Double-stranded ODNs were obtained by mixing equimolar amounts of complementary strands in the presence of 100 mM NaCl. We constructed pJCT, a plasmid containing the LTR-LTR junction, as follows: The HIV-1 LTR-LTR junction was amplified from HIV-1-infected CEM cells using gag 5′-gaattcgcgcttcagcaagccgagtc and env 5′-gaattcacccaaaaggtcagtgtggagtcc primers. The 1440-bp DNA fragment containing the LTR-LTR junction was digested with *Eco*RI and inserted into pCR2.1-TOPO (3.9 kb) (Invitrogen), yielding pJCT.

### IN purification and activity assays

The full-length HIV-1 WT IN, the 50-212 catalytic core domain (CC), the 1-212 two-domain protein (ΔC) and the E152A point mutant were prepared as previously described [21]. IN activity assays—3′-processing, disintegration and internal cleavage—were carried out at 37°C, in a buffer containing 10 mM HEPES (pH 7.2), 1 mM DTT, 7.5 mM MgCl_2_ or MnCl_2_ in the presence of 12.5 nM DNA substrate. Products were separated by electrophoresis in denaturing 18% acrylamide/urea gels. Gels were analysed with a Molecular Dynamics STORM phosphoimager and quantified with Image Quant™ 4.1 software. For palindrome cleavage, *Sca*I was used as a restriction control. *Sca*I recognises the 5′-AGTACT sequence and specifically cleaves after the 5′-GT.

For plasmid cleavage assays, pJCT containing the HIV LTR-LTR junction were incubated with 3 µM crosslinked IN oligomers for four hours, under conditions similar to those described for 3′-processing. Cleavage products were then analysed by electrophoresis in 1% agarose gels, with detection by BET staining. Crosslinked IN oligomers were produced and purified as previously described [10].

**Figure 2:**
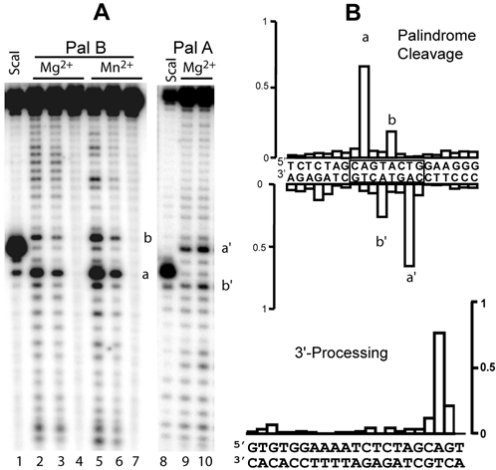
Endonucleolytic activity of HIV-1 IN on the palindromic U5-U3 junction. A) IN was incubated with 12.5 nM of 38-mer PalA/PalB duplex mimicking the HIV-1 palindromic U5-U3 junction in the presence of 7.5 mM divalent cation, for 2 h at 37°C. The 38-mer duplex was radiolabelled either on the 5′-extremity of the PalB ODN (top strand) (lanes 1-7) or on the 5′-extremity of the PalA ODN (bottom strand) (lanes 8–10). The metallic cofactor was Mg^2+^ (lanes 2–4, 9–10) or Mn^2+^ (lanes 5–7). Lanes 3, 6 and 9: 1.5 µM IN; Lanes 2, 5 and 10: 3 µM IN. Lanes 4 and 7: 3 µM IN + 1 mM EDTA. Lanes 1 and 8: PalA/PalB DNA substrate digested with *Sca*I. B) Relative cleavage efficiencies for the various DNA positions in the HIV-1 palindromic junction and 3′-processing substrate. The relative cleavage efficiency corresponds to a ratio between cleavage occurring at one position and total IN cleavage activity. This ratio is directly related to the specificity of the cleavage.

All experiments (activity and cross-linking experiments) were performed several times (up to five times each). Results were reproducible and each figure (Fig. 2–6) displays one representative experiment.

**Figure 3 pone-0000608-g003:**
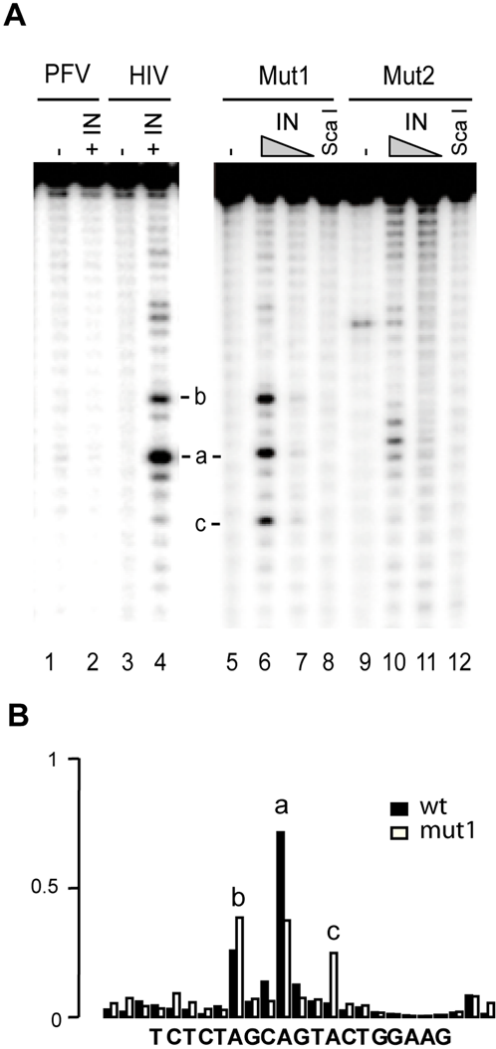
HIV-1 IN is highly specific for its cognate palindromic sequence and fails to cleave PVF and mutated HIV-1 palindromes. A) Cleavage activity of HIV-1 IN on PFV and HIV-1 palindromes. IN was incubated with DNA substrates in a Mg^2+^-containing buffer for 2 h at 37°C. Lanes 1 and 2: FP53B/FP53A substrate (PFV). Lanes 3 and 4: PalB/PalA substrate (HIV). Lanes 5–8: Mut1 substrate (CA->GT). Lanes 9–12: Mut2 substrate (GTAC->CATG). Lanes 2, 4, 6 and 10: 3 µM IN. Lanes 7 and 11: 1.5 µM IN. Lanes 1, 3, 5 and 9: negative control with 3 µM IN + 1 mM EDTA. Lanes 8 and 12: *Sca*I activity on mutated DNA duplexes. B) Histogram of cleavage efficiencies for the different DNA positions in the HIV-1 LTR-LTR junction. Black bars, wt. White bars, Mut1.

**Figure 4 pone-0000608-g004:**
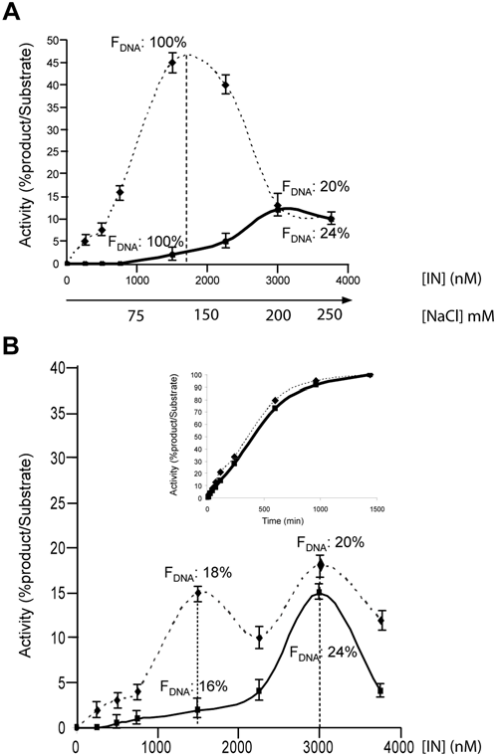
Differential responses of palindrome cleavage and 3′-processing activities to increasing IN concentrations. PalB/PalA or HIV38A/HIV38B duplexes (12.5 nM) were incubated with increasing concentrations of IN for 2 h at 37°C. Palindrome cleavage and 3′-processing activities were quantified as indicated in Materials and Methods and plotted versus IN concentration: Palindrome cleavage (straight line); 3′-processing (dashed line). A) Ionic strength increased with IN concentration. B) The experiment was performed as in A, except that ionic strength was kept constant ([NaCl]  =  200 mM final concentration). The time courses of product formation for palindrome cleavage and 3′-processing were compared under conditions of optimal IN concentration (3 µM), with 200 mM NaCl (inset). F_DNA_ represents the fractional saturation function of DNA sites, as measured by fluorescence anisotropy (see Materials and Methods), using either PalB/PalA or HIV38A/HIV38B duplexes. F_DNA_ is indicated for two IN concentrations (1.5 and 3 µM).

**Figure 5 pone-0000608-g005:**
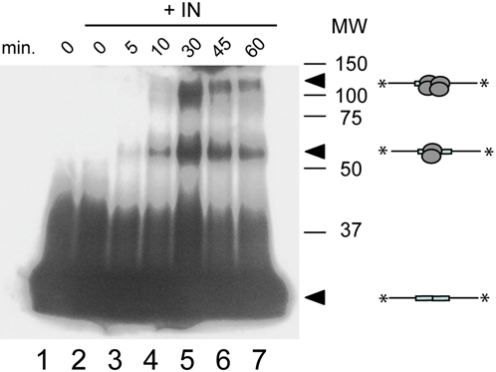
HIV-1 IN oligomer recruitment to the LTR-LTR junction. The 38-bp duplex PalB/PalA (1 pmol) was incubated with IN (5 pmol) for 0 to 60 min (lanes 2–7) in the presence of AHDAP (300 µM) in a final volume of 10 µL (yielding DNA and IN concentrations of 0.1 and 0.5 µM, respectively). Crosslinked products were then subjected to SDS-PAGE analysis and gel autoradiography. MW: Molecular weight markers (kDa). Lane 1: no IN. The weak reduction of the signal observed lanes 6 and 7 as comparison to the lane 5 is due to the time-dependent formation of higher-order oligomeric states of IN which are dependent on the AHDAP [10].

**Figure 6:**
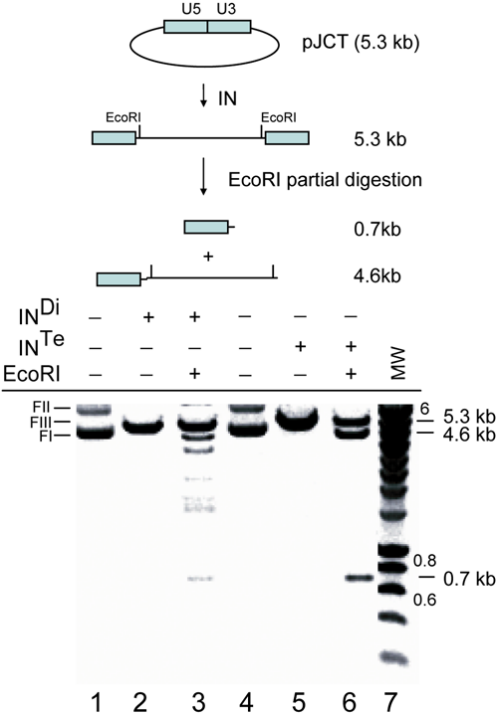
Palindrome cleavage by purified IN oligomers. The pJCT plasmid (12.5 nM) containing the LTR-LTR junction was incubated alone (lanes 1 and 4) or with 3 µM of either pure crosslinked dimers (Di) (lanes 2–3) or pure crosslinked tetramers (Te) of IN (lanes 5–6) for 4 hours at 37°C. It was then subjected to electrophoresis in an agarose gel (1%, 50V, 30 min) in the presence of BET (50 µg). Plasmid cleavage by IN was followed by partial digestion with *Eco*RI (lanes 3 and 6). MW: Molecular weight markers (kb).

### Chemical IN-DNA crosslinking

HIV-1 IN (5 pmol) was incubated with the PalA/PalB duplex (1 pmol) radiolabelled at its 5′-end in the presence of *cis*-aquahydroxydiamino platinum (AHDAP, 300 µM), 0.05% NP40, 10 mM DTT, 20 mM HEPES pH 7.5, at 37°C in the dark (final volume, 10 µl). The reaction was stopped by eliminating excess AHDAP, after crosslinking, by elution through a G25 MicroSpin column (Amersham) [10].

### Steady-state fluorescence anisotropy assay

Fluorescence anisotropy parameters were recorded with a Beacon 2000 instrument (PanVera, Madison, USA) in a cell maintained at 25°C. The formation of IN-DNA complexes was monitored by incubating fluorescein-labelled double-stranded ODNs with IN in 20 mM Tris (pH 7.2), 1 mM DTT, 100 or 200 mM NaCl, 5 mM MgCl_2_. The fractional saturation function of DNA (F_DNA_) was calculated as previously described [11], [22].

## Results

### HIV-1 WT specifically cleaves the U5-U3 palindromic junction in vitro

Analyses of HIV-1 integration sites have suggested that IN displays a preference for symmetric DNA sequences for the integration process [12]–[14]. We reasoned that IN might display specific recognition properties with DNA substrates containing symmetric or palindromic sequences, possibly leading to specific internal cleavage.

We tested this hypothesis, by assaying HIV-1 IN endonuclease activity in the presence of Mg^2+^ or Mn^2+^, using 38-bp ODNs containing several symmetric sequences in internal positions (Fig. 1B). We first tested the WP1 and WP2 palindromic sequences which mimic the weak palindrome consensus found at integration sites *in vivo* [12]–[14]. This sequence (..GTXAC..) displays an additional central base as compared to the palindromic LTR-LTR junction (Fig. 1B), where X represents any base with a bias toward A or T [12]. No significant cleavage was observed with WP1 and WP2 (data not shown). We next assessed the internal cleavage by IN of a palindromic 38-bp ODN that mimics the HIV-1 LTR-LTR junction. We observed a clear and reproducible cleavage of the palindromic sequence by radio-labelling the top strand (Pal B), regardless the divalent cation cofactor used (Fig. 2A; lanes 1–7) (no cleavage occurred in the absence of metallic cofactor or with single-stranded ODNs; data not shown). A control cleavage reaction with *Sca*I showed that cleavage occurred preferentially at a precise position downstream from the conserved 5′-CA sequence (primary site) matching the cleavage site observed for the standard 3′-processing of a single LTR extremity (band *a*). A weaker secondary cleavage site was also observed. This cleavage occurred reproducibly within the palindromic sequence after the next A residue in the 5′-3′ direction (band *b*). In addition to these two main sites, minor cleavage sites characteristic of the non-specific endonuclease activity of IN were also found throughout the length of the DNA.

We assessed the symmetry of specific cleavage by investigating IN activity on the other (bottom) strand of this palindrome (PalA; Fig. 2A, lanes 8–10). Again, specific cleavage was observed (bands a' and b'), with cleavage efficiency highest downstream from the 5′-CA position. Altogether, our data show that internal cleavage was clearly symmetric at the palindromic LTR-LTR junction. The efficiency of cleavage was dependent on the IN concentration for both strands. A better activity was obtained at 3 µM as compared to 1.5 µM (see also figure 4). The relative cleavage efficiencies for palindrome cleavage and 3′-processing reactions are indicated for each position in Fig. 2B. For these two reactions, the specific sites represent about 80% of the total IN activity on ODNs. Therefore, both reactions display similar reaction specificity. However, two major cleavage sites were observed in a reproducible manner for the palindrome cleavage and one site for the 3′-processing reaction. Moreover, it is important to note that efficiencies of 3′-processing and palindrome cleavage are comparable (see also the complete kinetics study in Fig. 4). Therefore, our results show that the cleavages occurring either at the DNA extremity or at the internal palindromic sequence are both specific and display similar catalytic efficiencies.

We then investigated the structural features of IN required for this activity, by testing the ability of point and deletion IN mutants to cleave the palindromic sequence. First, the catalytically inactive E152A mutant was unable to cleave the palindromic junction (Table I), regardless of the cationic cofactor used, showing that the DDE catalytic triad is directly involved in this cleavage. Second, we tested two IN mutants, one devoid of the C-terminal domain (ΔC) and the other, lacking both the N-terminal and the C-terminal domains (CC). These two deletion mutants were active in the disintegration assay in the presence of Mn^2+^ conditions (Table I), consistent with previous results [21], [23], [24]. However, they were not competent for palindrome cleavage (Table I). Thus, cleavage of the palindromic junction depends on the integrity of the full-length HIV-1 IN, closely paralleling the stringency observed for the 3′-processing reaction.

**Table 1:** Activities of the wild-type IN and mutants

	3′-processing^a^	Disintegration^b^	Palindrome cleavage^a^
WT IN	+	+	+
E152A	-	-	-
ΔC	-	+	-
CC	-	+	-

a Activities were assessed in the presence of either Mg^2+^ or Mn^2+^. ^b^ Disintegration activity was assessed in the presence of Mn^2+^, as previously described [21].

We then investigated whether the palindromic organisation of the DNA substrate containing the canonical 5′-CA dinucleotide was sufficient to ensure cleavage and assessed the tolerance to sequence changes of this cleavage. We recently showed that another retroviral IN, the primate foamy virus (PFV-1) IN, can cleave the palindromic sequence found at the PFV LTR-LTR junction [25]. We assessed the ability of HIV-1 IN to specifically cleave a palindromic sequence derived from the PFV LTR-LTR junction as well as several mutated versions of the HIV-1 palindromic sequence (Fig. 3A; see Fig. 1A for ODN sequences). The HIV-1 IN efficiently cleaved the eight-base HIV-1 palindromic sequence but did not cleave the six-base PFV 2-LTR junction which is specifically cleaved by the PFV-1 IN [25]. This suggests that retroviral INs display no cross-specificity for palindrome cleavage. We then investigated whether the four base pairs located between the canonical CA sequences of HIV-1 were required for cleavage using an inverted eight-base palindrome in which the four central bases were replaced by CATG (Mut2 substrate, see Fig. 1B). Again, no significant cleavage was detected, demonstrating that the palindromic organisation of the sequence was not sufficient for cleavage. We then investigated the role of the canonical 5′-CA, by replacing this dinucleotide with a 5′-GT sequence (Mut1 substrate, see Fig. 1B). Mut1 displayed clear cleavage sites within the boundaries of the palindrome, indicating that the 5′-CA is not crucial for the specific recruitment of IN to the viral substrate. However, cleavage levels were lower at the a position and higher at the b position than for the wild-type sequence (Fig. 3B). Interestingly, a third significant cleavage site was observed for Mut1 (band c), corresponding to a cleavage downstream from a 5′-TA sequence. This suggests that, in the absence of the canonical 5′-CA, IN probes the most favourable cleavage sites in the vicinity of the palindrome. Overall, our results show that palindrome cleavage has more or similar stringent requirements for the structural integrity of IN and viral DNA sequence than does the disintegration or the 3′-processing reactions, respectively.

### 3′-processing and palindrome cleavage require different oligomeric states

The 3′-processing activity of IN depends on the IN/DNA ratio, which controls the level of active complexes [11]. 3′-processing is optimal for IN/DNA ratio conditions corresponding to the dimerisation of IN on DNA [10], [11], [26]. We investigated the extent to which palindrome cleavage and 3′-processing could be compared, by determining the efficiencies of these processes as a function of IN concentration. The DNA substrates used were of identical size, to avoid a bias in IN binding due to oligonucleotide length [11]. We observed a typical bell-shaped curve for the 3′-processing reaction, and the optimal IN concentration was close to 1.5 µM (Fig. 4A, dashed line). We carried out fluorescence anisotropy experiments under these conditions and showed that all the DNA substrates in solution were bound by IN (F_DNA_  =  100%). In contrast to 3′-processing, palindrome cleavage activity was weak at 1.5 µM, although F_DNA_ was also 100%. However, more efficient palindrome cleavage was observed at higher protein concentrations, peaking at 3 µM IN (Fig. 4A, straight line). The amount of product obtained at this concentration was consistent with the fractional saturation function (F_DNA_  =  20%). The lower amount of active complexes can be fully explained by the higher ionic strength at the protein concentration of 3 µM ([NaCl]  =  200 mM). Taking into account the global effect of ionic strength, it is suggested that the activity per IN/DNA complexes is similar for palindrome cleavage and 3′-processing reactions. Consistently, both reactions display similar efficiencies for 3 µM IN concentration.

As ionic strength strongly influences the number of IN/DNA complexes, we next compared the palindrome cleavage and 3′-processing reactions in the presence of increasing IN concentrations, keeping salt concentration constant (200 mM) (Fig. 4B). With 1.5 µM IN, the efficiency of 3′-processing was about one third that obtained with the same IN concentration at lower ionic strength (compare Fig. 4A and 4B). This lower level of activity is due to the smaller number of complexes formed in the presence of 200 mM NaCl. Interestingly, the activity curve for 3′-processing was characterized by two maxima, one centred on 1.5 µM IN and the other centred on 3 µM (Fig. 4B). This suggests that 3′-processing occurs optimally with two types of complex, differing in the number of IN protomers complexed to DNA. In contrast, only the higher IN concentration allowed efficient palindrome cleavage, although similar numbers of IN/DNA complexes were obtained with the palindrome and the 3′-processing substrate (20% of complexes) for both IN concentrations (1.5 and 3 µM). Thus, only one type of complex is competent for palindrome cleavage and a low IN:DNA stoichiometry is not compatible with this reaction in contrast to what was observed for the 3′-processing reaction. In the second peak, corresponding to a high IN/DNA ratio, both reactions—3′-processing and palindrome cleavage—were equally efficient.

We compared the efficiency of the two reactions further at optimal IN concentration, by carrying out time-dependent experiments (Fig. 4B, inset). Both reactions were slow and linear over time for the first 10 hours, consistent with slow IN turnover [26]. In both cases, 90% of the product had been obtained by 16 hours of incubation. The kinetics of the two reactions were indistinguishable, confirming that palindrome cleavage is as efficient as 3′-processing.

Palindrome cleavage and 3′-processing responded differently to IN concentration, suggesting that these two reactions may be optimised by different oligomeric states of IN bound to DNA. We have previously shown that DNA-bound IN was mainly dimeric under conditions corresponding to the first peak of 3′-processing activity [11]. Here, we found that optimal palindrome cleavage activity as well as the second peak of 3′-processing were obtained with a higher IN concentration. Altogether, our results suggest that the dimeric IN catalyses the 3′-processing reaction but not the palindrome cleavage, whereas a higher-order multimeric state catalyses both reactions indifferently. It was previously shown that the decreasing phase observed for 3′-processing at high protein concentration is due to the formation of protein aggregates [11]. Most likely, protein aggregation onto DNA also accounts for the decreasing phases observed for the single peak of palindrome cleavage and the second peak of the 3′-processing reaction. For 3′-processing, we hypothesized that the decreasing phase observed between the two peaks of activity could be due to the formation of an intermediary oligomeric state, catalytically inactive.

### Palindromic DNA substrate recruits tetrameric IN

Recently, Faure *et al.* have identified by cross-linking analysis one IN dimer per LTR extremity and observed that a DNA fragment lacking the LTR sequence did not recruit discrete complexes [4], [10]. In this study, we investigated whether the presence of an internal LTR-LTR junction led to the recruitment or stabilisation of such discrete IN complexes using the same experimental approach. We carried out cross-linking experiments with a DNA fragment containing an internal HIV-1 LTR-LTR junction. This DNA fragment was radiolabelled, cross-linked to IN by incubation for various lengths of time and subjected to SDS-PAGE (Fig. 5). In contrast to what was observed for the one-LTR sequence, we found two cross-linked products with the LTR-LTR junction, corresponding to the dimeric and tetrameric forms of IN bound to DNA. Together with the activity results obtained, this experiment indicates that the LTR-LTR junction can recruit both multimeric forms—dimers and tetramers—although only the tetramer is competent for internal cleavage.

### Restriction-like activity of IN tetramers

Our results on short ODNs suggested that only tetramers were capable of cleaving the palindrome junction. We therefore investigated whether HIV-1 IN could cleave similarly longer DNA at internal positions. Purified covalent IN oligomers such as dimers and tetramers were obtained by chemically crosslinking and separated by size-exclusion chromatography, as previously described [10]. Dimers and tetramers were recovered and independently assayed for their ability to cleave a plasmid containing the palindromic LTR-LTR junction (the principle of the assay is explained in Figure 6). The incubation of IN dimers with DNA led to the accumulation of linear plasmid, indicating that double-strand cleavage had occurred (Fig. 6). After further digestion with *Eco*RI, we observed several bands, showing that the cleavage catalysed by the dimeric form occurred essentially non-specifically, at different sites in the DNA molecule. Linearisation of the plasmid was also obtained with IN tetramers, but further digestion with *Eco*RI yielded a single 700-bp fragment, showing that cleavage of a 5,300-bp plasmid had occurred, in a restriction-like manner, at a single position corresponding to the LTR-LTR junction. The specific cleavage at the LTR-LTR junction also occurs with dimers but this cleavage is rather weak as compared to the cleavage observed with the tetrameric IN. This could be explained either by a weak palindrome cleavage activity of dimers or a significant formation of tetramers (i.e. non-covalent dimer of dimers) which are competent for palindrome cleavage.

We also assessed the palindrome cleavage activity of purified tetramers, dimers and monomers, using the 38-bp DNA substrate PalA/PalB. Again, the efficiencies of the various forms of IN differed—tetramers (50% cleavage)>dimers (3% cleavage)>monomers (no cleavage) (data not shown)—confirming that the tetrameric form was the most efficient for internal cleavage. In conclusion, our results provide for the first time the demonstration that HIV-1 is highly competent to cleave the LTR-LTR junction.

## Discussion

We have identified a new type of endonuclease activity of HIV-1 IN that requires the enzyme to be organised as a tetramer on a specific DNA sequence. IN specifically cleaves, in a reproducible manner, DNA at internal positions, only if the cleavage site is formed by the palindromic sequence corresponding to the HIV-1 LTR-LTR junction. IN has been reported to cleave internal sequences in several studies [27]–[29]. However, the reported activity was weak and non-specific in all these cases. In particular, truncated IN proteins (CC, ΔC, ΔN) inactive in 3′-processing or strand transfer retain this endonuclease activity [30]. In contrast, the palindromic junction cleavage described here is highly sequence-specific, requires the full-length protein and is efficient with the physiologically relevant cofactor, Mg^2+^. Palindrome cleavage also differs markedly in this respect from the disintegration reaction that can be performed by CC, ΔC or ΔN truncated proteins only in the presence of Mn^2+^, or by the full-length IN with a large preference for Mn^2+^ over Mg^2+^ [23], [24].

Internal cleavage mostly occurs at the 5′-CA position corresponding to the CA of the 3′-processing reaction, symmetrically on the minus and plus strands. We also identified a secondary cleavage site downstream from the 5′-CA, at the 5′-TA sequence. Interestingly, mutation of the 5′-CA canonical dinucleotide revealed a supplementary cleavage at the 5′-TA sequence upstream from the mutated position, suggesting that cleavage may be delocalised, occurring at adjacent sites in the absence of the canonical 5′-CA. Studies of the conformational properties of nine different dinucleotides for which adequate data from crystal structures were available indicated that CA was by far the most flexible, resulting in its selection at transposon termini due to its significant conformational mobility [31]. The TA dinucleotide is the next most flexible [32]. It has been suggested that this flexibility of the CA dinucleotide is critical for DNA melting/distortion events before cleavage, making it possible for this strand to be engaged by the active site of the MuA transposase. These observations suggest that IN recruitment to the DNA substrate may be followed by a probing phase enabling IN to find a favourable site for cleavage—5′-CA or 5′-TA sequences. According to this model, the presence of the canonical 5′-CA in the WT palindrome would restrict upward movement, whereas movement in both directions is possible if this sequence is missing.

Using purified cross-linked IN multimers, we showed that although LTR-LTR junctions recruited both the dimeric and tetrameric forms of IN, only tetramers specifically and efficiently cleaved the junction. Early studies reported that the avian sarcoma virus integrase could cleave the LTR-LTR junction [33], [34]. However, this result was not reproduced with the HIV-1 IN [35]. Our results suggest that this may be due to the stringent conditions for palindrome cleavage, which is strictly dependent on the ability of HIV-1 IN to form an active tetrameric enzyme. This may account for the low efficiency or absence of palindrome cleavage at IN concentrations maximising the 3′-processing of short ODNs, which is known to occur with a dimeric enzyme [10], [11].

The requirement of a tetrameric form for palindrome cleavage parallels the results recently described for concerted integration showing that a dimer of dimers allows insertion of the two DNA ends into two different strands of the target DNA [3]–[5], [14]. However, the weak palindromic consensus which has been observed at the integration sites *in vivo* [12]–[14] differs from the LTR-LTR junction by an additional central base. Hence, we did not observe a specific cleavage with DNA substrates mimicking this consensus sequence, indicating that the additional central base is a specific feature of integration sites. Moreover, our results indicate that the cleavage of the palindromic LTR-LTR junction is not tolerant to sequence modifications and thus is a highly specific reaction, whereas the integration sites, characterized *in vivo*, only exhibit a weak consensus. The symmetrical consensus, found at integration sites *in vivo*, means that a palindromic sequence in the DNA target favours the transesterification reaction (nucleophilic attack conducted by the 3′-OH viral DNA extremity) resulting in the full integration process. Our assay does not mimic a strand transfer (or integration) assay but rather reveals endonucleolytic reactions (nucleophilic attack mediated by a water molecule). This means that the specific cleavage of the LTR-LTR junction is primarily mediated by a water molecule and the catalytic mechanism of IN for this cleavage is similar to the well-known mechanism for 3′-processing. The catalytic reaction is more specific in term of sequence when a water molecule is involved in the nucleophilic attack, as observed for 3′-processing [6], [7] and 2-LTR junction cleavage (this study) as compared to reactions directly involving the terminal 3′-hydroxyl of the viral DNA as a nucleophilic agent (half transfer [21] and the full site integration in symmetrical sequences [12]–[14]). These results suggest that both palindrome cleavage and integration require a tetrameric form but are based on different mechanisms. Nevertheless, both the strict dependence on palindromic sequences for internal cleavage and the palindromic bias of the integration demonstrate that tetrameric IN intrinsically prefers to bind to symmetric DNA sites.

We recently reported an equivalent activity for PFV-1 IN with its cognate palindromic substrate. These observations suggest that palindrome cleavage may be an intrinsic property of retroviral INs [25]. The strong specificity of such symmetric internal cleavage and the fact that the only palindrome cleaved corresponded to the cognate LTR-LTR junction naturally present in infected cells, within 2-LTR circles, suggest that palindrome recognition by IN may play an important role at different stages of the replication cycle. *In vitro*, internal cleavage at the HIV-1 LTR-LTR junction mostly produces extremities with a four-base overhang, different from the two-base overhang generated by 3′-processing. No such intermediate DNA product has yet been identified in infected cells, in contrast to the canonical 3′-processing product, which constitutes the precursor of the integrated provirus. Thus, in the absence of further cellular evidence, it remains unclear whether the palindrome cleavage of LTR-LTR junctions contributes to the overall equilibrium of the different forms of viral DNA in the cell.
